# Therapeutic benefit of the dual ALK/FAK inhibitor ESK440 in ALK-driven neuroblastoma

**DOI:** 10.1016/j.neo.2024.100964

**Published:** 2024-01-06

**Authors:** Seema Chugh, Jean C. Tien, Jennifer Hon, Carson Kenum, Rahul Mannan, Yunhui Cheng, Chi Chiang Li, Zainab I. Taher, Andrew D. Delekta, Pushpinder Singh Bawa, Ingrid J. Apel, Stephanie J. Miner, Xuhong Cao, Rohit Mehra, Saravana M. Dhanasekaran, Yuanyuan Qiao, Rajen Mody, Arul M. Chinnaiyan

**Affiliations:** a Michigan Center for Translational Pathology, University of Michigan, Ann Arbor, MI 48109, USA; b Department of Pathology, University of Michigan, Ann Arbor, MI 48109, USA; c Department of Pediatrics, University of Michigan, Ann Arbor, MI 48109, USA; d Howard Hughes Medical Institute, University of Michigan, Ann Arbor, MI 48109, USA; e Department of Urology, University of Michigan, Ann Arbor, MI 48109, USA; f Rogel Cancer Center, University of Michigan, Ann Arbor, MI 48109, USA

**Keywords:** Neuroblastoma, ALK, FAK, ESK440, MYCN

## Abstract

Neuroblastoma (NB) is a predominantly pediatric cancer with greater than 90% of cases arising in children under the age of five. More than half of patients have metastases detected at diagnosis, and high-risk disease is associated with five-year survival rates of only 50–60 %. Standard therapy involves highly toxic chemotherapy, surgery, radiation, and immunotherapy, and less toxic, more specific targeted therapies are urgently needed. Genomic studies have identified common driver aberrations in high-risk NB, such as *MYCN* amplification. In addition, a proportion of high-risk patients harbor amplification or activating mutations in anaplastic lymphoma kinase (*ALK*), and co-occurrence of *ALK* mutations and *MYCN* amplification have been associated with aggressive disease. In this study, we analyzed the efficacy of a Phase Ia-cleared, orally bioavailable dual ALK and focal adhesion kinase (FAK) inhibitor, ESK440, in multiple preclinical NB models. ESK440 potently inhibited proliferation of NB cell lines, with increased sensitivity in cell lines harboring *ALK* aberrations. ALK, FAK, and downstream target activation were rapidly decreased upon ESK440 treatment, and this was associated with impaired cellular migration and invasion. Importantly, ESK440 treatment also decreased MYCN levels. NB cell line and patient-derived xenograft studies showed significant reduction in tumor growth in ESK440-treated mice with no signs of toxicity. In certain NB models, ESK440 showed comparable or enhanced efficacy to lorlatinib, another clinical ALK inhibitor, and a lorlatinib-resistant cell line (COG-N-561 LR) retained sensitivity to ESK440. These preclinical results indicate that ESK440 is a promising targeted agent for ALK-driven NB and support future clinical studies to evaluate its efficacy in NB patients.

## Introduction

Neuroblastoma (NB) is the most common extracranial pediatric solid tumor and accounts for ∼15% of cancer-related deaths in children [1]. NB arises from primitive nerve cells, with primary tumors often developing in the adrenal medulla or other nerve tissue near the spine or in the abdomen and chest [2]. Patients presenting with low-risk NB have an encouraging prognosis, with five-year survival rates for this group at more than 95 % [3]. However, over half of NB patients have metastatic disease at diagnosis and are stratified as high-risk neuroblastoma (HR-NB), and the outlook for these patients is less promising [3,4]. While targeted therapies have been effective at treating the advanced states of several adult cancers, the development of targeted therapy for treating HR-NB is still in the very early stages —primarily because the hallmark genomic aberration of HR-NB, *MYCN* amplification, has been difficult to target [5]. In addition to *MYCN* amplification, ∼12–15 % of NB patients harbor genetic aberrations in anaplastic lymphoma kinase (*ALK*). These aberrations have been associated with poor survival, and the majority (85 %) occur at three positions — R1275, F1174, and F1245 [6,7]. Studies have observed the co-occurrence of *ALK* mutations with *MYCN* amplifications and have suggested a synergistic effect on oncogenesis [8,9].

While the FDA-approved ALK inhibitor crizotinib has been successfully used to target ALK fusions in lung cancer, Phase I clinical studies using crizotinib saw only a modest response in NB patients, and preclinical studies with crizotinib demonstrated reduced sensitivity in a NB model with F1174L-mutated ALK [10,11]. Further, the compound demonstrated poor blood-brain penetration, one of the known challenges in targeting brain metastasis in relapsed NB cases [12]. Although several other next-generation ALK inhibitors, including lorlatinib, are being evaluated in clinical trials based on promising preclinical data, the highly heterogeneous nature of the disease and development of resistance to ALK inhibitor therapies underscores the need for novel, complex therapeutic strategies [13], [14], [15].

To overcome some of the clinical challenges and to target this aggressive pediatric malignancy, we have evaluated therapeutic efficacy of a compound called ESK440 (formerly known as CEP-37440) that is a dual inhibitor of ALK and focal adhesion kinase (FAK) [16]. ALK and FAK are multi-functional tyrosine kinases that play essential roles in neuronal development [17,18]. Several genomic aberrations of *ALK* (overexpression, mutations, and amplification) have been described in NB [6,19,20]. Furthermore, FAK has been associated with increased metastasis and is found to be overexpressed in *MYCN*-amplified NB [15,21]. However, the therapeutic potential of dual ALK and FAK inhibition has not been fully explored in NB.

ESK440 is the only known dual ALK and FAK inhibitor that inhibits the enzymatic and cellular biochemical activity of both ALK and FAK at low nanomolar concentrations [16]. The half maximal inhibitory concentration (IC_50_) values were previously shown to be 3.1 and 2.0 nM in enzyme-based *in vitro* assays, and 22 and 82 nM in corresponding cell-based biochemical assays for wild-type ALK and FAK, respectively [16]. The orally bioavailable compound has cleared a Phase Ia clinical trial (NCT01922752) in adult subjects and can bind several mutated forms of ALK. The Phase Ia trial determined a maximum tolerated dose (MTD) of 500 mg/day for ESK440, with nausea being the dose-limiting toxicity in all cases. Additionally, the compound can penetrate the blood-brain barrier, one of the major limitations in targeting relapsed NB. Here, we wanted to determine the anti-cancer efficacy of ESK440 in NB models and provide a preclinical rationale for testing ESK440 in NB patients with *ALK* genomic aberrations.

## Materials and Methods

### Cell lines

Most neuroblastoma cell lines were obtained from the Childhood Cancer Repository (https://www.cccells.org/). Diffuse intrinsic pontine glioma (DIPG) cells were generously provided by Dr. Michelle Monje (Stanford University) and Dr. Sriram Venneti (University of Michigan). The remaining cancer cell lines were procured from ATCC. Recommended guidelines were used to culture these cancer cells from different lineages. Cell lines were genotyped and were confirmed to be mycoplasma-free before carrying out any functional assays.

### Compounds

ESK440/CEP-37440 was provided by Esanik Therapeutics, which licensed the compound from Teva Pharmaceuticals. Defactinib (Cat.No-S7654), crizotinib (Cat.No-S1068), and lorlatinib (Cat.No-S7536) were purchased from Selleckchem.

### Cell viability and dose-response assay

1000-5000 cells were seeded in 96-well white color plates (Fisher Scientific). Twenty-four hours after plating, a serial dilution of ESK440 was prepared and added to the plate (6 replicates per dose). The cells were incubated for six days, and cell viability was assessed using Cell-Titer Glo assay (Promega). GraphPad Prism was used to calculate IC_50_ values.

### Incucyte proliferation assay

Impact of ESK440 on cell proliferation was assessed using the IncuCyte live-cell imaging system. Drug dose-response assays were carried out in 96-clear well plates as described above. Following drug addition, plates were kept in IncuCyte chambers.

### Crystal violet staining assay

1000-5000 cells were seeded in 96-well clear plates, and long-term survival assays were performed after 10–14 days of ESK440 treatment. Following long-term incubation, plates were fixed with 4 % formaldehyde, stained with 1 % crystal violet, and imaged.

### Western blotting

NB cells were treated with different drugs at several time points. Following drug incubation, cells were washed with PBS and lysed with 1X RIPA buffer. Lysates were quantified, and western blotting was performed using several antibodies. The antibodies used were pFAK-Y397 (Cell Signaling Technology 3283S), total FAK (Cell Signaling Technology 3285S), pALK-Y1604 (Cell Signaling Technology 3341S), total ALK (Cell Signaling Technology 3633S), pERK (Cell Signaling 4376S), total ERK (Cell Signaling Technology 9102S), pAkt (Cell Signaling Technology 4060S), and total Akt (Cell Signaling Technology 9272S). Imaging was performed using LICOR Odyssey M.

### Migration and invasion assays

NB cell suspension with DMSO control and several concentrations of ESK440 was placed in 8 μm Matrigel-coated (invasion) and non-coated (migration) Fluoroblok chambers (Corning). Media with 10 % FBS was placed in the lower chamber (chemoattractant). 24 h after incubation, migrated and invaded cells were stained with calcium AM green, and relative migration/invasion was quantified with a TECAN M1000 fluorescent plate reader.

### *In vivo* studies

Male CB17 SCID mice (6–8 weeks of age) were used for all experiments. The anti-tumor efficacy of ESK440 was evaluated in one NB cell line (NB-1, ATCC) and three patient-derived xenograft (PDX) models (Felix, COG-N-452, COG-N-453; all from Childhood Cancer Repository). NB-1 xenografts were generated by subcutaneous injection of Matrigel-suspended NB-1 cells (5  ×  10^6^) into both flanks. PDX xenografts were generated by subcutaneous implantation of tumor chunks into both flanks. In each case, xenograft growth was monitored twice weekly by caliper measurement for the duration of the experiment. Once tumors reached ∼80–100 mm^3^, mice were randomized (*n* = 10/group) to receive ESK440 (60mg/kg or 90 mg/kg) or PEG-400 vehicle (5 days/week) by oral gavage for 14–30 days. At the end of the time course, tumors were excised, weighed, measured, photographed, fixed, and paraffin embedded for histologic analysis. All animal work was approved by the Institutional Animal Care and Use Committee of the University of Michigan.

### Immunohistochemistry (IHC)

Tissue sections from *in vivo* pharmacodynamic and therapeutic studies were formalin-fixed and embedded in paraffin blocks. The primary antibodies used were pALK-Y1604 (Invitrogen, 1:100), pFAK-Y397 (Cell signaling Technology, 1:100), MYCN (Santa-Cruz), and Ki-67 (Biocare Medical, 1:100).

#### IHC scoring

Ki-67: For Ki-67, the number of tumor nuclei which had positive nuclear expression for the protein were calculated in a total of 500 cells from representative areas under a bright field microscope. The final score in the end was expressed as /100 cells (percentage positive cells).

MYCN: The presence and intensity of MYCN nuclear staining were scored by the study pathologists where the percentage of MYCN-positive tumor cells and the staining intensity (none, 0; weak, 1; moderate, 2; strong, 3) were recorded for each tumor as described previously [22]. We then compared the distribution of individual scores of IHC (0, 1, 2, and 3) in the vehicle and treated tumors as a performance indicator (to characterize the degree of uniformity of score distribution) by calculating the average percentage of cells scored 1, 2, and 3 for MYCN IHC for both cohorts, normalizing the total score to 100 % (as described above) to construct the representative pie charts in Fig. 3C (for easier data visualization).

### Blood chemistry analysis

Blood from vehicle and ESK440 treated mice were collected using BD Microtainer SST tubes. The samples were centrifuged at 7000 rpm for 10 min, and serum was isolated. Serum samples were given to the Unit for Laboratory Animal Medicine Pathology Cores (ULAM) for Animal Research for assessment of renal and liver chemistry.

### Development of lorlatinib-resistant NB models

The IC_90_ dose of lorlatinib was used to develop drug-resistant NB models. COG-561 NB cells were cultured in lorlatinib-containing (10 μM) media for a few weeks that resulted in elimination of lorlatinib-sensitive NB cells and subsequent enrichment of a resistant cell population.

## Results

### ESK440 exhibits preferential cytotoxicity in NB cells with *ALK* genomic aberrations

**Fig. 1:**
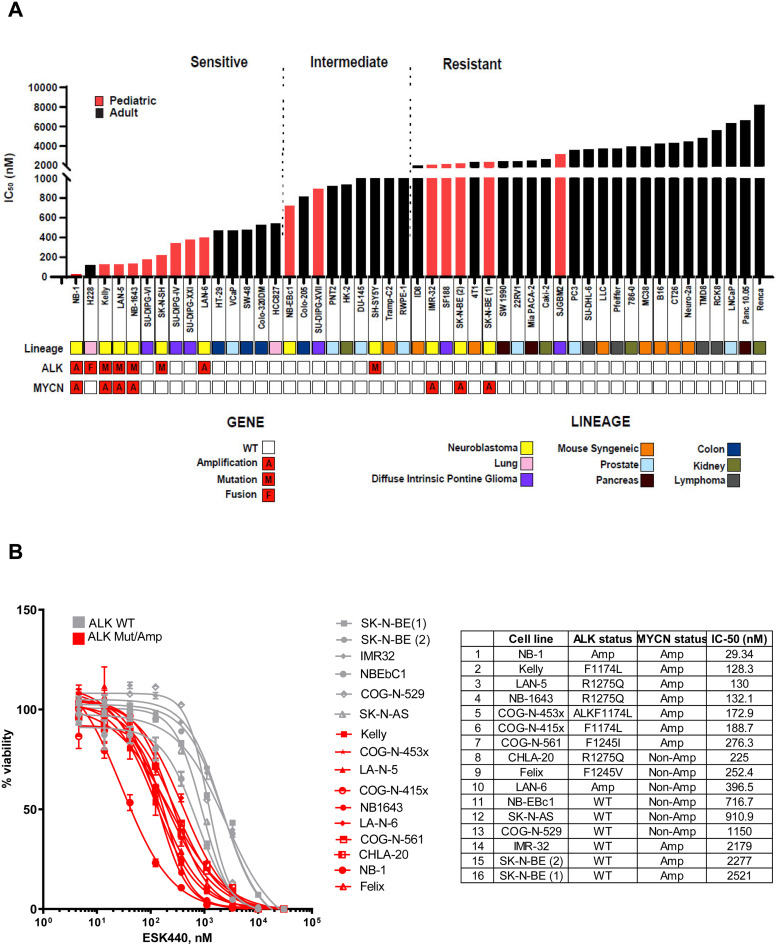
Neuroblastoma (NB) cells with *ALK* genomic aberrations are preferentially sensitive to ESK440. (A) A panel of cell lines across different lineages were analyzed for growth sensitivity to ESK440 (1 week), and IC_50_ values were plotted. (B) Dose response curves for NB cell lines (*n* = 16) treated with ESK440 (1 week). Table shows *ALK* and *MYCN* status as well as IC_50_ values.

The cellular response to treatment with ESK440 was characterized in a large cohort comprising a multi-cancer cell line panel consisting of several human cancer cell lines representing diverse cancer types (*N* = 49). Cell lines were ranked by IC_50_ values and divided into sensitive, intermediate, and resistant groups (Fig. 1A). The pattern of sensitivity was notable in cancer cell lines with *ALK* fusions, mutations, and amplifications. Some of the diffuse intrinsic pontine glioma (DIPG) cell lines also showed increased sensitivity. Non-small cell lung cancer (NSCLC) cells with *EML4-ALK* fusions (H228) showed sensitivity (IC_50_=118.4 nM). Interestingly, several NB cell lines showed increased sensitivity (Fig. 1A). Most of the remaining cancer cell lines from other malignancies such as pancreas, lung, kidney, and lymphoma remained resistant.

Dose-response curves of 16 NB cell lines indicated increased sensitivity of NB cells with *ALK* amplifications and mutations in comparison to NB cells with wild-type *ALK* (Fig. 1B). NB-1 cells with *ALK* amplification were highly sensitive with an IC_50_ value of 29.34 nM. ESK440 sensitivity was noted across different *ALK* mutations such as F1174L, R1275Q, F1245I, and F1245V (Fig. 1B). Incucyte assays and crystal-violet staining further confirmed the dose-dependent decrease of cell viability in selected sensitive models (Supplementary Fig. 1A and B).

### ESK440 inhibits ALK and FAK-associated signaling pathways in NB cells

**Fig. 2:**
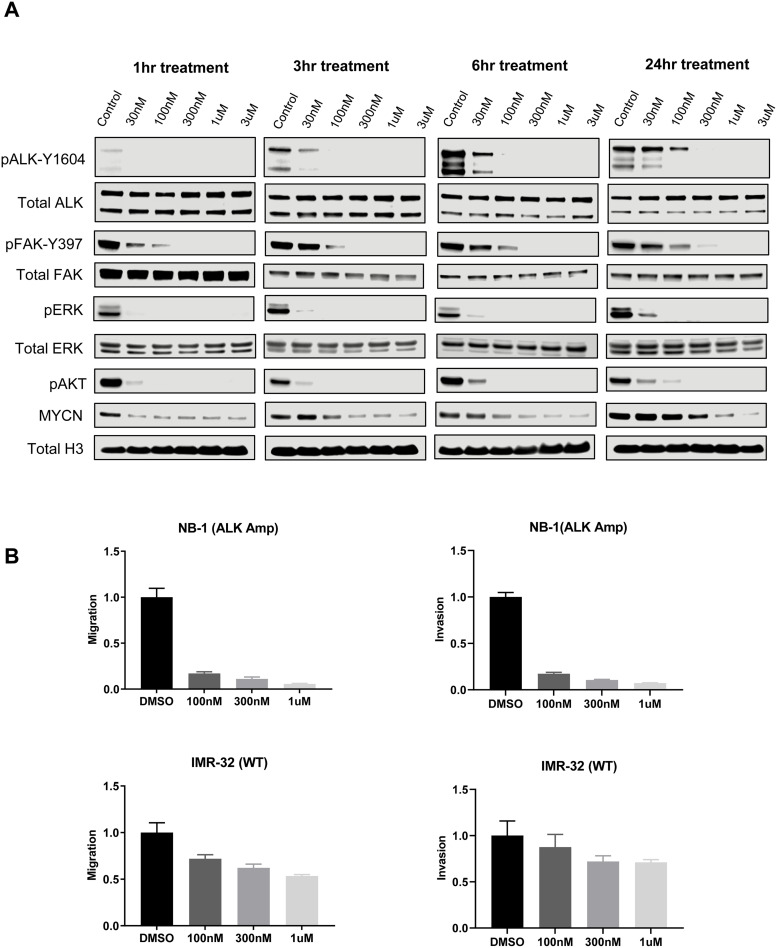
ESK440 inhibits ALK and FAK-associated signaling pathways. (A) NB-1 cells were treated with increasing concentrations of ESK440 at different time points. Cell lysates were processed for western blot analysis of the indicated proteins. Total Histone H3 serves as a loading control. (B) Migration and invasion in *ALK*-amplified or *ALK* wild-type (WT) NB cell lines after 24 h of treatment (*n* = 3 technical replicates).

To investigate the effects of ESK440 on ALK and FAK activation and downstream signaling pathways, we performed western blotting in NB-1 cells treated with increasing doses of ESK440 at different time points (Fig. 2A). We observed dose-dependent decreases in phosphorylated ALK (pALK-Y1604) and FAK (pFAK-Y397) at all time points tested. The decreases in activated ALK and FAK were more pronounced at early time points (1 h, 3 h, 6 h) in comparison to the late time point (24 h). Similarly, we observed dramatic decreases of downstream effector AKT signaling pathway activation at early time points. However, there was a remarkable reduction in pERK signaling at all time points tested. Interestingly, we also noted decreased MYCN protein expression upon treatment with increasing doses of ESK440, which was most prominent at early time points. We further validated this in Kelly NB cells (*ALK* F1174L mutation) and observed dose-dependent decreases of ALK and FAK activation (Supplementary Fig. 2).

Since FAK activation has been implicated in increased migration and metastasis, the impact of ESK440 on migration and invasion of NB cell lines was evaluated (Fig. 2B). Migration and invasion assays were carried out in NB-1 cells (*ALK* amplification) and IMR-32 cells (wild-type *ALK*) for 24 h. Significantly decreased migration and invasion were noted in *ALK*-amplified NB-1 cells at all doses, whereas ESK440 had less significant effects on migration and invasion of IMR-32 cells expressing wild-type *ALK*.

### ESK440 inhibits ALK and FAK activation *in vivo*

**Fig. 3:**
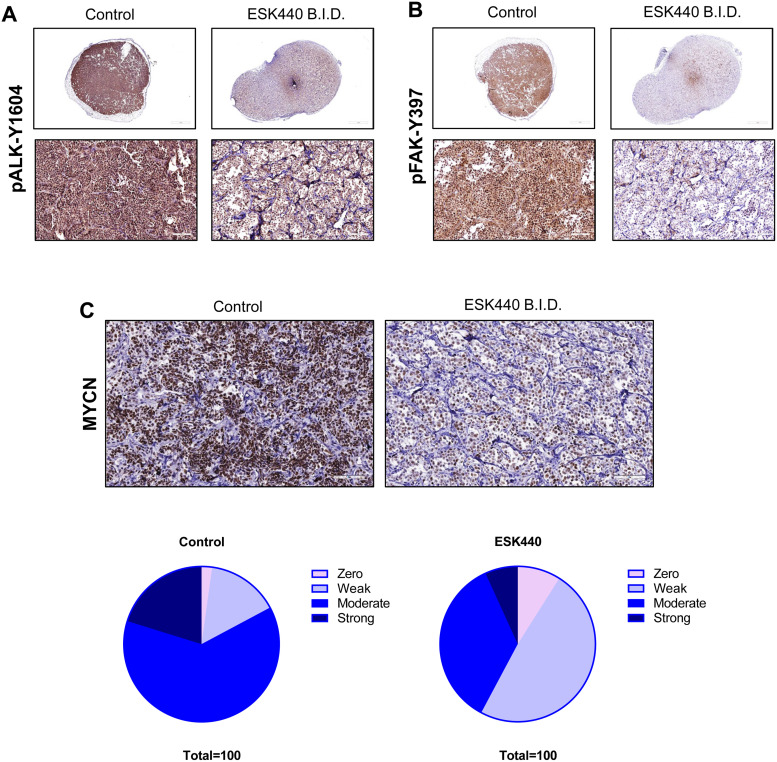
ESK440 decreases ALK and FAK phosphorylation *in vivo* as well as MYCN levels. Mice bearing NB-1 cell line-derived xenografts were treated with ESK440 at 60 mg/kg BID for 1 week. Immunohistochemistry of tumors harvested after sacrifice showed decreased levels of (A) phospho-ALK, (B) phospho-FAK, and (C) MYCN.

To ascertain the primary target selectivity of ESK440, the *in vivo* pharmacodynamic activity of ESK440 was assessed. Mice bearing NB-1 xenograft tumors were treated with ESK440 60 mg/kg (QD and BID, PO) for 5 days. Tumor tissues were harvested 4 h after the last treatment, and activation states of ALK and FAK were evaluated using immunohistochemistry (IHC) and western blotting. Dose-dependent inhibition of both targets (pALK-Y1604, pFAK-Y397) was observed in the xenograft model systems, as demonstrated in IHC images (Fig. 3A and B) and western blotting data (Supplementary Fig. 3). Notably, MYCN protein levels were also decreased in tumors following ESK440 treatment (Fig. 3C).

### ESK440 inhibits tumor growth in multiple xenograft models of neuroblastoma

**Fig. 4:**
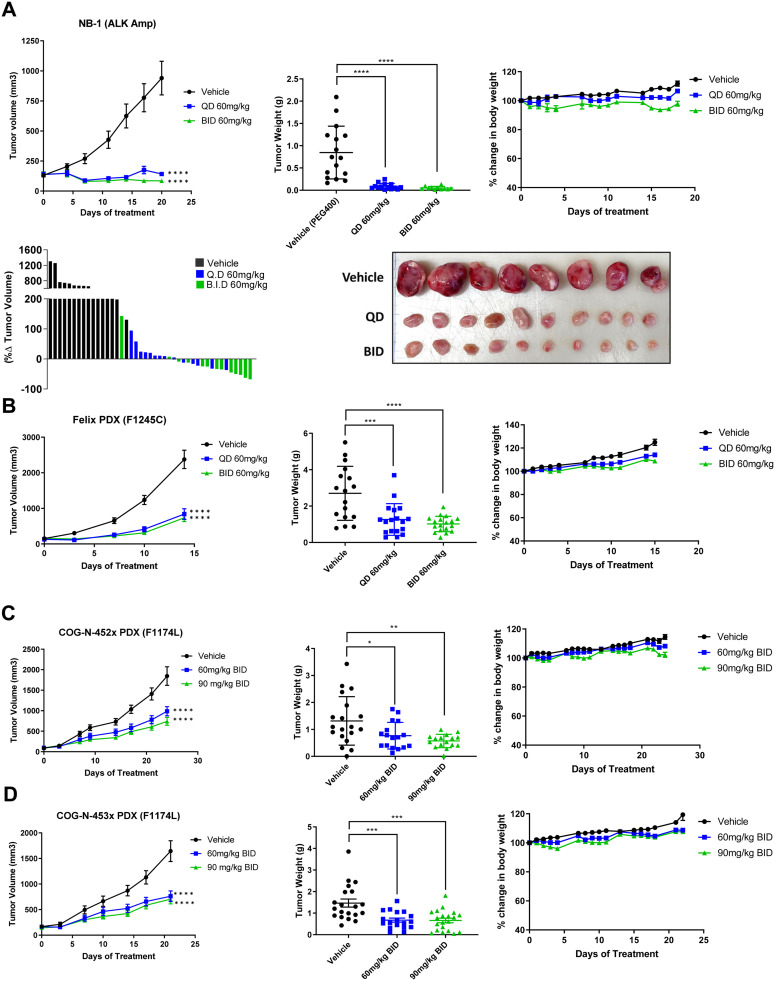
ESK440 decreases tumor growth in NB xenograft models. ESK440 efficacy study in (A) NB-1 model, (B) Felix patient-derived xenograft (PDX), (C) COG-N-452 PDX, and (D) COG-N-453 PDX. Tumor volume, tumor weight, and body weight changes were recorded (left to right). ****P < 0.0001, ***P < 0.0005, **P < 0.005, and *P < <0.05 (one-way ANOVA for tumor weights, two-way ANOVA for tumor volumes).

**Fig. 5:**
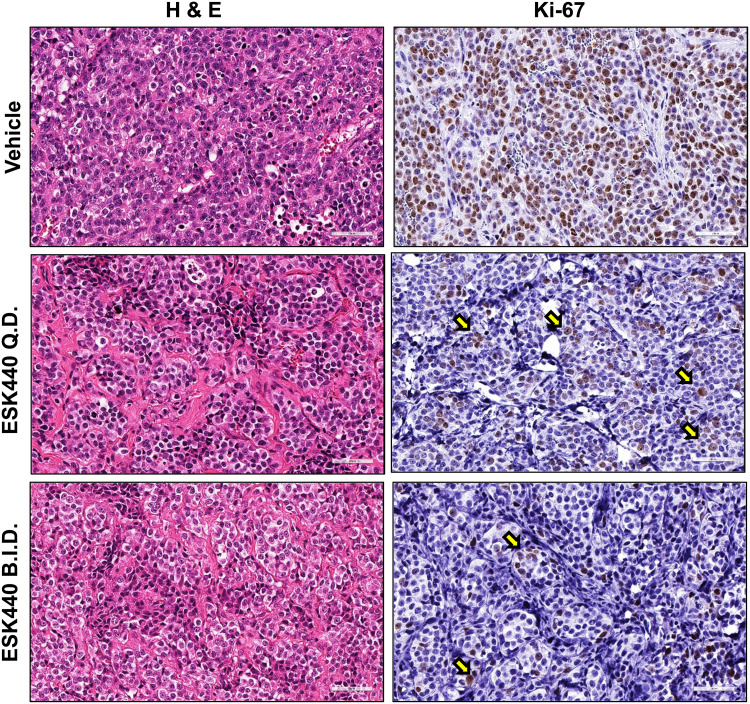
ESK440 decreases tumor cell proliferation. Representative H&E images and Ki-67 immunohistochemistry of NB-1 tumors treated for four weeks with vehicle or the indicated regimen of ESK440 (60 mg/kg QD or BID).

To further assess the anti-tumor effect of ESK440, therapeutic studies were performed in several xenograft models of NB. Initial studies were carried out using the NB-1 cell line xenograft model, which carries *ALK* and *MYCN* amplification and showed enhanced sensitivity to ESK440 *in vitro* (Fig. 1A and B). ESK440 was administered at 60 mg/kg QD and BID in NB-1 xenografts for four weeks. ESK440 treatment with 60 mg/kg QD PO resulted in significant inhibition of tumor growth and tumor weights (Fig. 4A); administration of ESK440 at 60 mg/kg BID also resulted in significant tumor regression and a dramatic decrease in tumor weights. Immunohistochemistry analysis showed markedly reduced Ki-67 positive tumor cells in QD and BID treatments in comparison to vehicle controls (Fig. 5, Supplementary Fig. 4A).

The efficacy study was further conducted in multiple PDX models of NB. ESK440 efficacy was evaluated in the Felix NB model that harbors the *ALK* F1245C mutation (WT *MYCN*) and is associated with crizotinib resistance [23]. QD and BID oral administration of ESK440 at 60 mg/kg over a period of two weeks significantly decreased tumor weights and volumes (Fig. 4B). Next, the therapeutic efficacy of ESK440 was evaluated in COG-N-452x and COG-N-453x, both of which exhibit *ALK* 1174L mutation and *MYCN* amplification (resistance to crizotinib) [24]. In the COG-N-452x PDX model, both QD and BID administration were effective in significantly inhibiting tumor volume and tumor weights (Fig. 4C). ESK440 treatment also inhibited tumor growth in the COG-N-453x PDX model at both dosing regimens (Fig. 4D). Importantly, ESK440 treatment did not lead to significant body weight alterations in any of the xenograft models tested (Fig. 4A–D). Further, no specific toxicities in liver, renal, or other functions were noted (Supplementary Fig. 4B). Collectively, these preclinical studies in several xenograft models with different *ALK* aberrations demonstrate that ESK440 treatment significantly impacts tumor growth kinetics in ALK-driven NB models.

### Comparison of ESK440 with lorlatinib in neuroblastoma models

**Fig. 6:**
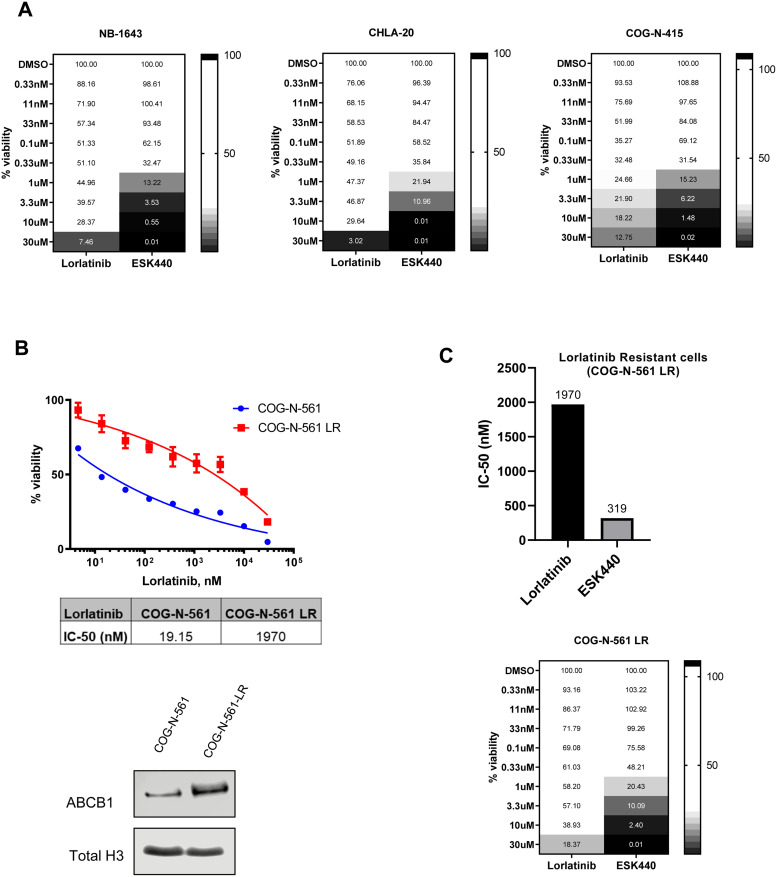
ESK440 exhibits enhanced efficacy in certain NB cell lines, and lorlatinib-resistant cell lines remain sensitive to ESK440. (A) Cell viability plots for the indicated NB cell lines treated for 1 week with ESK440 or lorlatinib. (B) Lorlatinib-resistant (LR) COG-N-561 cells were established and treated with increasing doses of lorlatinib (top graph), and lorlatinib IC_50_ values for the LR and parental cell line were calculated (bottom table). Western blot showing increased level of ABCB1 in LR cell line compared to parental; total histone H3 serves as a loading control. (C) COG-N-561 LR cells were treated with ESK440 or lorlatinib for 1 week, and the IC_50_ values (top graph) and percent viabilities (bottom) were determined.

We next compared the efficacy of ESK440 with the next-generation ALK inhibitor lorlatinib/PF-06463922 that is undergoing Phase I clinical trials in NB (NCT03107988) [25]. ESK440 was compared with lorlatinib across several NB cellular models with different types of *ALK* mutations (ALK F1174L, F1245V, 1275Q) and *ALK* amplification. IC_50_ analysis indicated potent activity of lorlatinib in these models with IC_50_ values ranging from 1-18 nM in highly sensitive cell lines (Supplementary Fig. 5A and B). In NB models with R1275Q mutation (CHLA-20, NB-1643), IC_50_ values of ESK440 were similar to lorlatinib (Supplementary Fig. 5A and B). We also compared ESK440 activity with crizotinib (ALK inhibitor) and defactinib (FAK inhibitor). While ESK440 displayed similar activity as crizotinib in NB cell models, ESK440 was much more potent than defactinib in killing NB cells (Supplementary Fig. 5B). Intriguingly, across all models tested, ESK440 dose-escalation resulted in complete cell death; however, a fraction of NB cells survived high doses of lorlatinib treatment (Fig. 6A, Supplementary Fig. 5C). In many of the models, approximately 18–30 % of cells remained viable even with 10 μM of lorlatinib (Fig. 6A). We further developed a lorlatinib-resistant NB cell line (COG-N-561 LR) and found increased ABCB1 protein expression (Fig. 6B). This suggests the potential involvement of ABCB1 (MDR1/P-Gp) in mediating acquired resistance to lorlatinib. ABCB1/P-glycoprotein is an ATP-dependent efflux pump, which has been associated with multidrug resistance [26,27]. Interestingly, ESK440 was able to efficiently kill lorlatinib resistant cells (COG-N-561 LR) with an IC_50_ value of 319 nM. In the COG-N-561 LR cell line, increased doses of ESK440 achieved complete cell death (Fig. 6C).

## Discussion

Genomic aberrations in *ALK* were first described in NB in 2008 [6,19,20,28]. Since then, multiple efforts have been directed to understand the functional implications of different ALK mutations and therapeutic targeting of this protein in the setting of HR-NB [29]. Crizotinib (a competitive inhibitor of ALK and MET kinase) was the first ALK inhibitor that was FDA-approved for metastatic ALK-positive NSCLC. Following FDA approval in NSCLC, clinical trials with crizotinib were also initiated in ALK-positive NB (ADVL0912, ANBL1531) [30]. However, the results from the Phase II studies with crizotinib were less promising where a response rate of only 15 % was seen in refractory or relapsed ALK-mutant NB patients [30]. In parallel, several other next-generation inhibitors have also been assessed that resulted in amendment of the ANBL1531 trial, where crizotinib was replaced by the third-generation ALK inhibitor, lorlatinib [29]. Lorlatinib (ALK and ROS inhibitor) has shown potent therapeutic efficacy in several preclinical models of NB [13,31]. However, resistance to lorlatinib has been seen in NSCLC and is also anticipated in NB patients [32,33]. Further, lorlatinib treatment resulted in several central nervous system (CNS) effects such as seizures and altered cognitive functions [34]. This necessitates the need to develop more advanced ALK inhibitors to overcome drug resistance and toxicity.

In the current study, we characterized the preclinical therapeutic efficacy of the compound ESK440, which is an orally active small-molecule ALK and FAK inhibitor that has completed a Phase Ia clinical trial in adult subjects with a favorable safety profile (no neurotoxicity reported) (NCT01922752). Further, this compound has excellent metabolic activity, a promising pharmacokinetic profile, binds several mutated forms of ALK, and can penetrate the blood-brain barrier. Using an *in vitro* drug screening approach, we found that ESK440 significantly inhibits proliferation of cancer cells with different types of *ALK* genomic aberrations such as lung cancer cells (*EML4-ALK* fusion) and NB cells (mutations, amplifications), as well as some of the DIPG cells (Fig. 1). Based on this *in vitro* screen of ESK440 and the lack of therapies efficiently targeting NB, we focused our mechanistic studies and preclinical evaluation on models of NB. ESK440 treatment in NB cell lines potently inhibits ALK and FAK phosphorylation and is associated with marked inhibition of signaling pathways such as AKT, ERK, and MYCN that have been implicated in NB growth (Fig. 2-3). Our preclinical studies using cell line-derived xenografts and several PDX models provide compelling data on the ant-tumor activity of ESK440 in ALK-driven NB models (Figs. 4 and 5). Furthermore, the lack of evident toxicity in the animal models tested and the favorable safety data from the Phase Ia clinical trial suggest that ESK440 would be well-tolerated in the setting of NB patients. However, it is important to note that patients in the Phase Ia trial were all over the age of 18, whereas the majority of NB cases are in pediatric patients.

We further compared the efficacy of ESK440 with lorlatinib across multiple NB cell line models due to the clinical interest in this inhibitor. While lorlatinib is extremely potent across many NB models, ESK440 displayed similar cytotoxic activity as lorlatinib in some of the R1275Q-mutated cell line models such as NB-1643 and CHLA-20. Lorlatinib treatment did not result in complete cell death in most NB cells, even at high doses, whereas ESK440 treatment effectively killed all cells (Fig. 6). Mechanisms of lorlatinib resistance are not yet well-defined in NB. Our study suggests increased P-gp/ABCB1 expression as one of the mechanisms of lorlatinib resistance in NB. Overexpression of ABCB1 has been associated with drug resistance [26,27]. Interestingly, ESK440 demonstrated cytotoxic activity in lorlatinib-resistant NB cells (Fig. 6C). Future efforts will be aimed at comprehensively characterizing lorlatinib resistance mechanisms in NB and evaluating the impact of ESK440 monotherapy, as well as combination with chemotherapy and/or immunotherapy, in lorlatinib-resistant NB. Taken together, our study suggests the potential clinical utility of ESK440 in ALK-driven NB.

## CRediT authorship contribution statement

**Seema Chugh:** Conceptualization, Data curation, Formal analysis, Investigation, Visualization, Writing – original draft, Writing – review & editing. **Jean C. Tien:** Conceptualization, Data curation, Formal analysis, Investigation, Visualization, Writing – review & editing. **Jennifer Hon:** Investigation. **Carson Kenum:** Investigation. **Rahul Mannan:** Investigation. **Yunhui Cheng:** Investigation. **Chi Chiang Li:** Investigation. **Zainab I. Taher:** Investigation. **Andrew D. Delekta:** Investigation. **Pushpinder Singh Bawa:** Formal analysis. **Ingrid J. Apel:** Investigation. **Stephanie J. Miner:** Writing – original draft, Writing – review & editing. **Xuhong Cao:** Investigation. **Rohit Mehra:** Investigation. **Saravana M. Dhanasekaran:** Investigation. **Yuanyuan Qiao:** Investigation. **Rajen Mody:** Conceptualization, Supervision, Writing – review & editing. **Arul M. Chinnaiyan:** Conceptualization, Funding acquisition, Writing – original draft, Writing – review & editing.

## Declaration of competing interest

The authors declare the following financial interests/personal relationships which may be considered as potential competing interests:

The University of Michigan has filed a provisional patent application based on the findings from this study (A.M.C. and S.C. are named as co-inventors). A.M.C. is a co-founder and serves on the scientific advisory board of Esanik Therapeutics, Inc. that licensed ESK440 from Teva Pharmaceuticals. Teva Pharmaceuticals or Esanik Therapeutics did not contribute to study design and funding. A.M.C. is also a co-founder and scientific advisor to LynxDx, Medsyn Bio, and Flamingo Therapeutics. He serves as an advisor to RAAPTA Therapeutics, Ascentage Pharma, Aurigene Oncology, and Tempus. The remaining authors declare no conflict of interest.

## Appendix Supplementary materials

Image, application 1

Image, application 2
