# Supplementary material for: Therapeutic benefit of the dual ALK/FAK inhibitor ESK440 in ALK-driven neuroblastoma
Source: Neoplasia. 2024 Jan 6;60:100964. doi: 10.1016/j.neo.2024.100964 (PMC11846495; doi:10.1016/j.neo.2024.100964)
Supplement: Supplementary file 1 [file mmc1.docx]

**Supplementary Figure Legends**

**Supplementary Fig. 1.** **ESK440 inhibits proliferation of NB cells harboring *ALK* aberrations** **(A)** Incucyte growth curves of IMR-32 (*ALK*-WT) and NB-1 (*ALK*-Amp) cells treated with control and increasing concentrations of ESK440 for 5-7 days (n=6 technical replicates, mean ± s.e.m are shown). **(B)** Long-term cell viability assessment by crystal violet staining in indicated NB cells treated with increasing concentrations of ESK440 (n=3 technical replicates).

**Supplementary Fig. 2. ESK440 inhibits ALK and FAK signaling in NB cells.** Immunoblot analysis of pFAK-Y397, total FAK, pALK-Y1604, total ALK, pERK, and total ERK in Kelly cells treated with increasing concentrations of ESK440 for 24hr.

**Supplementary Fig. 3. Pharmacodynamic profiling of ESK440 in NB-1 tumor xenograft model. (A)** Immunoblot analysis of pFAK-Y397, total FAK, and MYCN in tumor tissue lysates collected from mice treated with vehicle control and ESK440 for 5 days. **(B)** Immunoblot analysis of pALK-Y1604 and total ALK in tumor tissues collected from mice treated with vehicle control and ESK440 for 5 days.

**Supplementary Fig. 4. Evaluation of ESK440-treated NB-1 tumor xenografts. (A)** Quantification of Ki-67 staining in control and ESK440-treated NB-1 xenograft tumors (n=5/group). **(B)** Blood chemistry of renal, liver, and other functions in mice treated with vehicle control and ESK440 (n=6-8 mice/group).

**Supplementary Fig. 5. Comparison of ESK440 efficacy with other ALK inhibitors in NB cell lines. (A)** IC-50 comparison of ESK440 and lorlatinib in COG-N-415, Felix, CHLA-20, and COG-N-529 NB cells. **(B)** IC-50 comparison of ESK440 (ALK/FAK inhibitor), lorlatinib (ALK inhibitor), crizotinib (ALK inhibitor), and defactinib (FAK inhibitor) in NB-1 and NB-1643 cells. **(C)** Comparison of percentage viability of Felix and NB-1 cells treated with increasing doses of ESK440 and lorlatinib (n=6 technical replicates).
