# Supplementary material for: Therapeutic benefit of the dual ALK/FAK inhibitor ESK440 in ALK-driven neuroblastoma
Source: Neoplasia. 2024 Jan 6;60:100964. doi: 10.1016/j.neo.2024.100964 (PMC11846495; doi:10.1016/j.neo.2024.100964)

Supplementary Figure 1

A

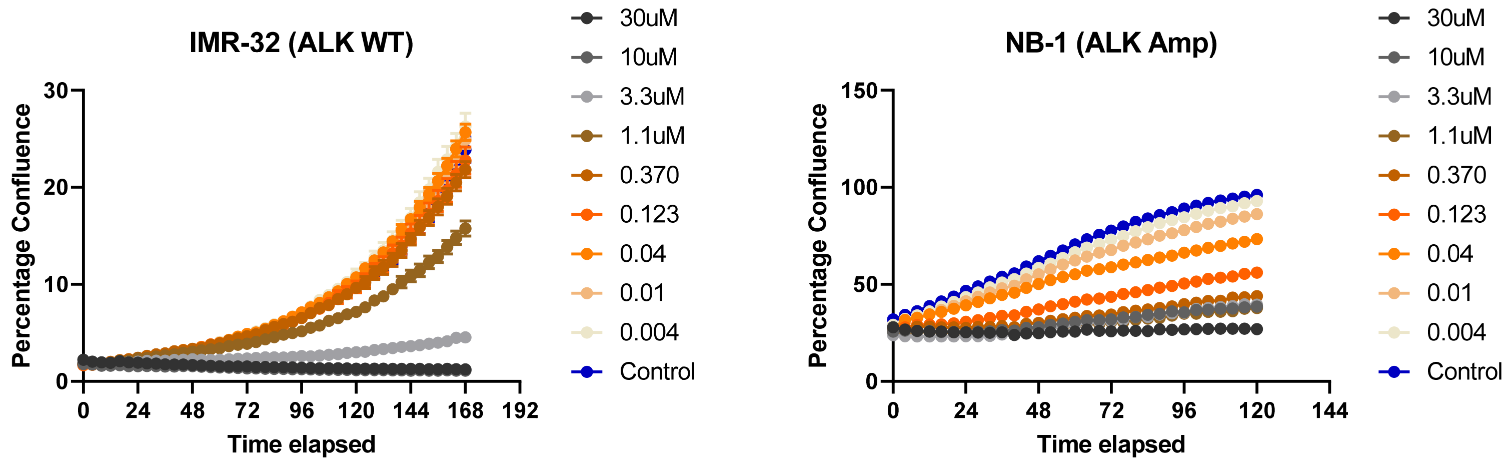

B

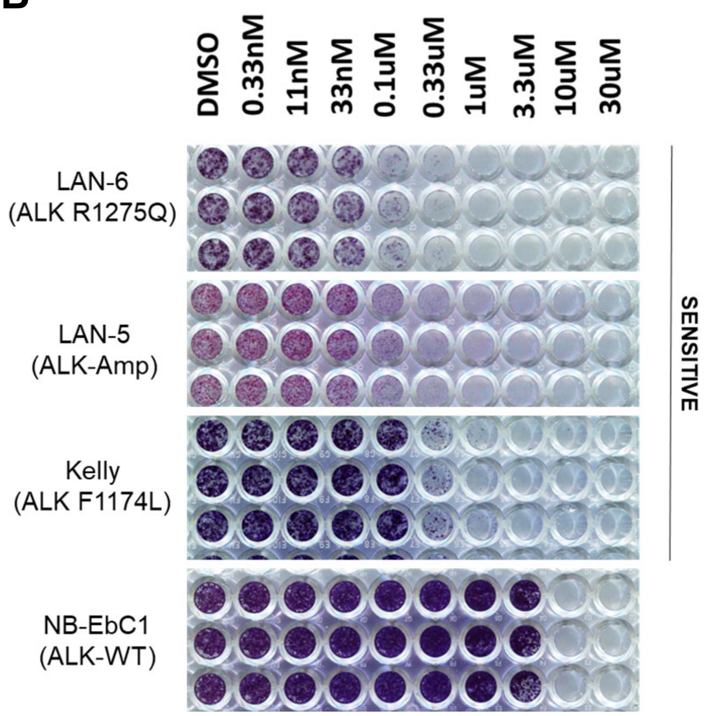

Supplementary Figure 2

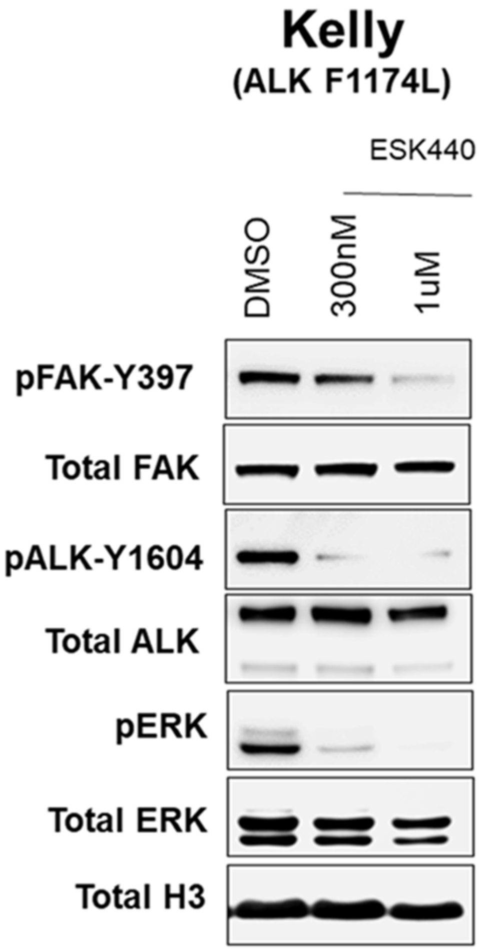

Supplementary Figure 3

A

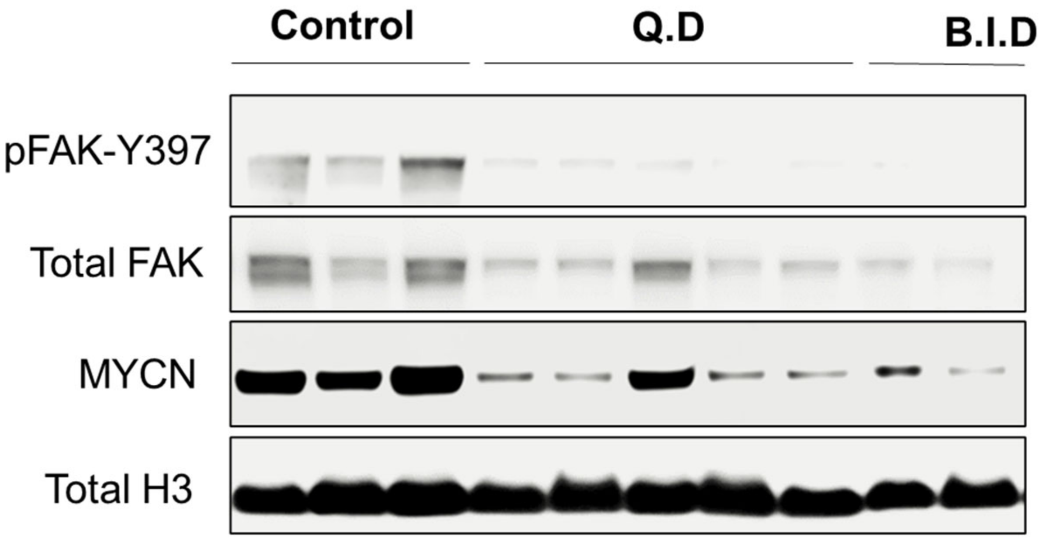

B

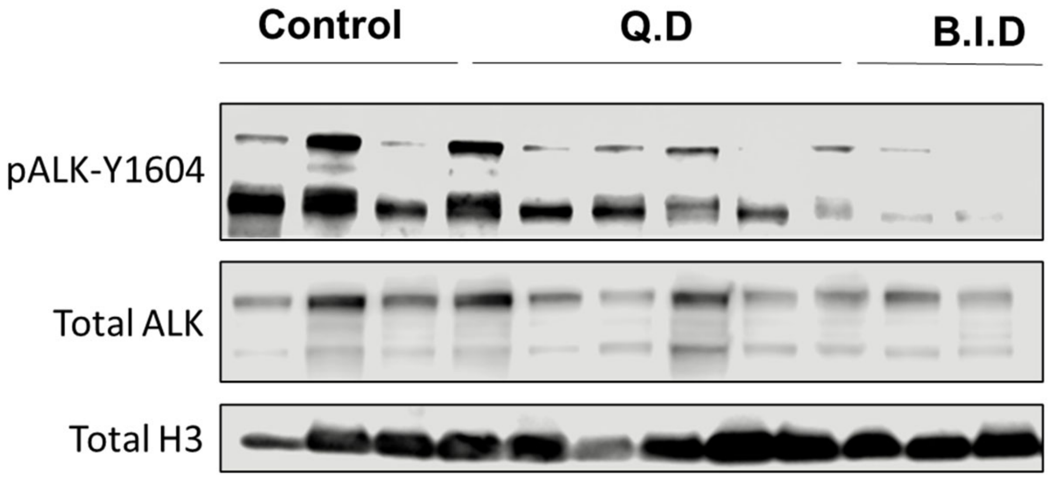

Supplementary Figure 4

A

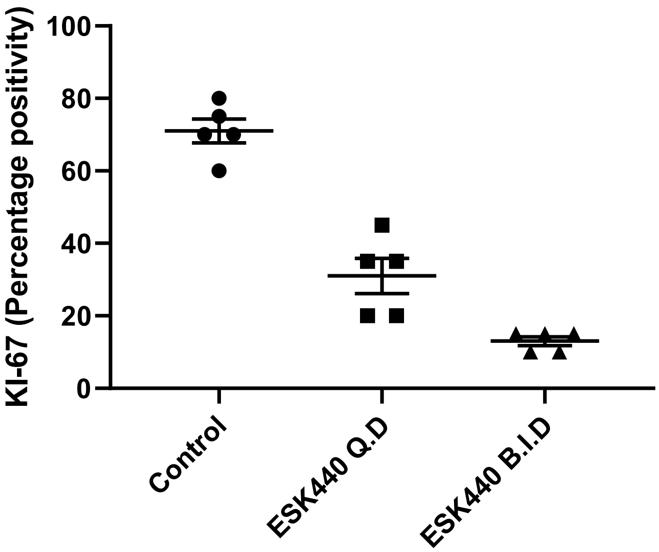

B

		Vehicle	QD	BID	Normal range	Unit
LIVER FUNCTION	ALKP	65.1	96.7	96.9	65.5-364.2	U/L
	ALT	64.28	65.19	67.00	24.3-115.25	U/L
	Tbili	0.22	0.22	0.30	0.12-0.58	mg/dL
RENAL FUNCTION	BUN	24.1	23.5	23.3	5.15-30.7	mg/dL
	CREA	0.33	0.39	0.43	0.09-0.4	mg/dL
OTHER	Ca	10.18	10.10	9.78	9.03-12.4	mg/dL
	Gluc	141.88	159.57	194.17	79.35-354.73	mg/dL

Supplementary Figure 5

A

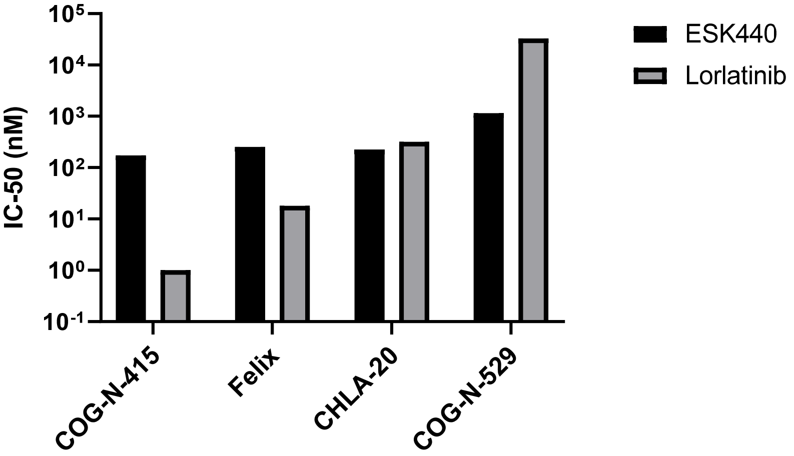

Cell line	ALK	ESK440 (nM)	Lorlatinib (nM)
COG-N-415	F1174L	172	1
Felix	F1245V	252	18
CHLA-20	R1275Q	225	319
COG-N-529	WT	1150	32891

B

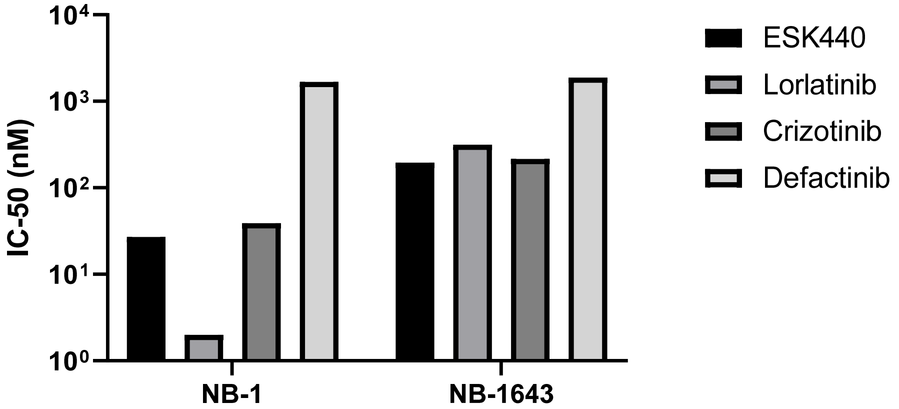

Cell line	ESK440	Lorlatinib	Crizotinib	Defactinib
NB-1	27	2	39	1681
NB-1643	195	316	216	1877

C

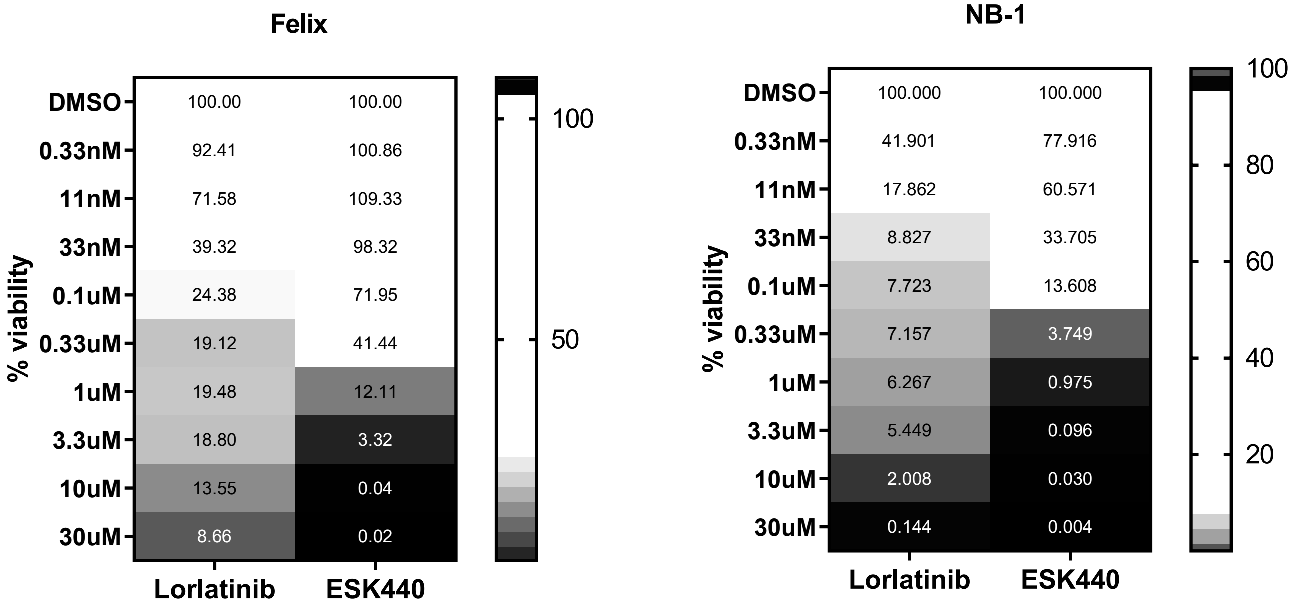

Supplement: Supplementary file 2 [file mmc2.pdf]
